# Optimal Iodine Staining of Cardiac Tissue for X-Ray Computed Tomography

**DOI:** 10.1371/journal.pone.0105552

**Published:** 2014-08-29

**Authors:** Timothy D. Butters, Simon J. Castro, Tristan Lowe, Yanmin Zhang, Ming Lei, Philip J. Withers, Henggui Zhang

**Affiliations:** 1 School of Physics and Astronomy, The University of Manchester, Manchester, Greater Manchester, United Kingdom; 2 School of Materials Science, The University of Manchester, Manchester, Greater Manchester, United Kingdom; 3 Institute of Cardiovascular Sciences, Faculty of Medical and Human Sciences, The University of Manchester, Manchester, Greater Manchester, United Kingdom; 4 Department of Pharmacology, University of Oxford, Oxford, Oxfordshire, United Kingdom; Illinois Institute of Technology, United States of America

## Abstract

X-ray computed tomography (XCT) has been shown to be an effective imaging technique for a variety of materials. Due to the relatively low differential attenuation of X-rays in biological tissue, a high density contrast agent is often required to obtain optimal contrast. The contrast agent, iodine potassium iodide (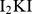), has been used in several biological studies to augment the use of XCT scanning. Recently 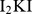 was used in XCT scans of animal hearts to study cardiac structure and to generate 3D anatomical computer models. However, to date there has been no thorough study into the optimal use of 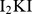 as a contrast agent in cardiac muscle with respect to the staining times required, which has been shown to impact significantly upon the quality of results. In this study we address this issue by systematically scanning samples at various stages of the staining process. To achieve this, mouse hearts were stained for up to 58 hours and scanned at regular intervals of 6–7 hours throughout this process. Optimal staining was found to depend upon the thickness of the tissue; a simple empirical exponential relationship was derived to allow calculation of the required staining time for cardiac samples of an arbitrary size.

## Introduction

Imaging the heart is of vital importance to the study of cardiac anatomy and the role of structural remodelling in various pathologies, including heart failure. Cardiac imaging also lends itself to investigating the role of structure in excitation wave conduction, and it's contribution to arrhythmogenesis. Over the years various techniques have been used to map cardiac structures at a range of spatial scales. These vary from magnetic resonance imaging (MRI) for whole organ and tissue section scanning, to scanning electron microscopy (SEM) to elucidate sub-cellular structures of cardiac myocytes [1], [2]. At the whole organ and tissue section level the reconstructions can be used to study structural differences between healthy and diseased organs, as well as for computational simulation studies in which the electrical activation of the heart is modelled in a realistic geometry [3]–[6]. For both of these applications a high resolution reconstruction with a fine level of detail is required to resolve structures that play a role in the development of cardiac disease [7], [8].

For many years MRI has been the ‘gold standard’ for heart imaging, especially when reconstructing the whole organ. However, due to the limitations of sample size within large magnetic field systems it has only been possible to scan the hearts of small animals such as mice, rats and rabbits at a high resolution (∼50* µ*m) [9]. Reconstructions of larger samples, such as those from humans, have been limited to a voxel size of 1–5 mm for 1.5–3.0 T clinical systems [10].

X-ray computed tomography (XCT) has many advantages over MRI for cardiac reconstructions. The sample size is limited only by the bay in which the scanner is housed; it is therefore possible to scan hearts from large mammals as well as those from small animals such as mice with the same machine. Voxel sizes of ∼40* µ*m and ∼36* µ*m have previously been obtained in rat [11] and dog [12] hearts respectively; since resolution scales with sample size, we would expect a resolution of ∼100* µ*m could be obtained for the human heart. The scan times are also typically much shorter with XCT, taking anywhere from a few minutes [13], [14] to ∼2 hours, compared to ∼10 hours for a comparable MRI scan [15].

Unfortunately, XCT images of biological tissue tend to be of low contrast, greatly limiting the use and application of standard XCT results. Several studies have shown however, that by using a suitable contrast agent there is an increase in differential attenuation of X-rays and contrast is improved. Metscher has highlighted several contrast agents for use in biological samples, including iodine potassium iodide (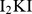) and phosphotungstic acid, all of which improve contrast and allow various structures to be easily identified [16]. More recently, Jeffery *et al.* were able to resolve the arrangement of muscle fibres in rodent heads with application of 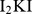 [17]. In a similar study, Stephenson *et al.* were able to obtain sufficient contrast in rat and rabbit hearts with 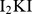 so as to segment the cardiac conduction system [11], [12].

The staining procedure is essential in order to achieve feature definition during the reconstruction and segmentation stages. The amount of time the tissue is incubated in the staining solution is a key factor for contrast, as both under- and over-staining have negative consequences. If the tissue is prematurely removed from the solution the staining will be incomplete and parts of the tissue will retain their low contrast properties when imaged. This can complicate analysis; for example, automated methods of segmentation, such as region-growing, often rely on each distinct region having consistent intensity. Automatic methods of segmentation fail as a result of incomplete staining, increasing the need for manual segmentation. Conversely, staining tissue with 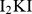 for prolonged periods of time has been shown to cause tissue shrinkage; whilst the extent of shrinkage can be reduced using a suitable fixative, it cannot be completely eliminated [18]. Therefore, an optimal staining time would be the minimum time required to completely stain the sample, reducing the effects of shrinkage as much as possible. However, this can only be determined by trial and error, and, to the best of our knowledge, there is no study which quantifies the staining process and its relationship to sample size.

This study aims to address this issue by quantifying the staining process and identifying the optimal incubation time for cardiac tissue in 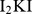 solution for XCT imaging. To achieve this, mouse hearts were scanned at a series of staining stages to track the saturation of solution within the tissue. From the resulting data it was possible to deduce a relationship between the tissue thickness and required staining time, which will allow one to determine the optimal staining time for a given sample size.

## Materials and Methods

### Ethics Statement

This study was approved by the University Research Ethics Committee, University of Manchester. The animals from which samples were obtained were euthanized in accordance with directive 2010/63/EU of the European Parliament and in compliance with the ethical standards of the University of Manchester and the UK Home Office Animals (Scientific Procedures) Act 1986.

### Mouse Hearts

**Figure 1:**
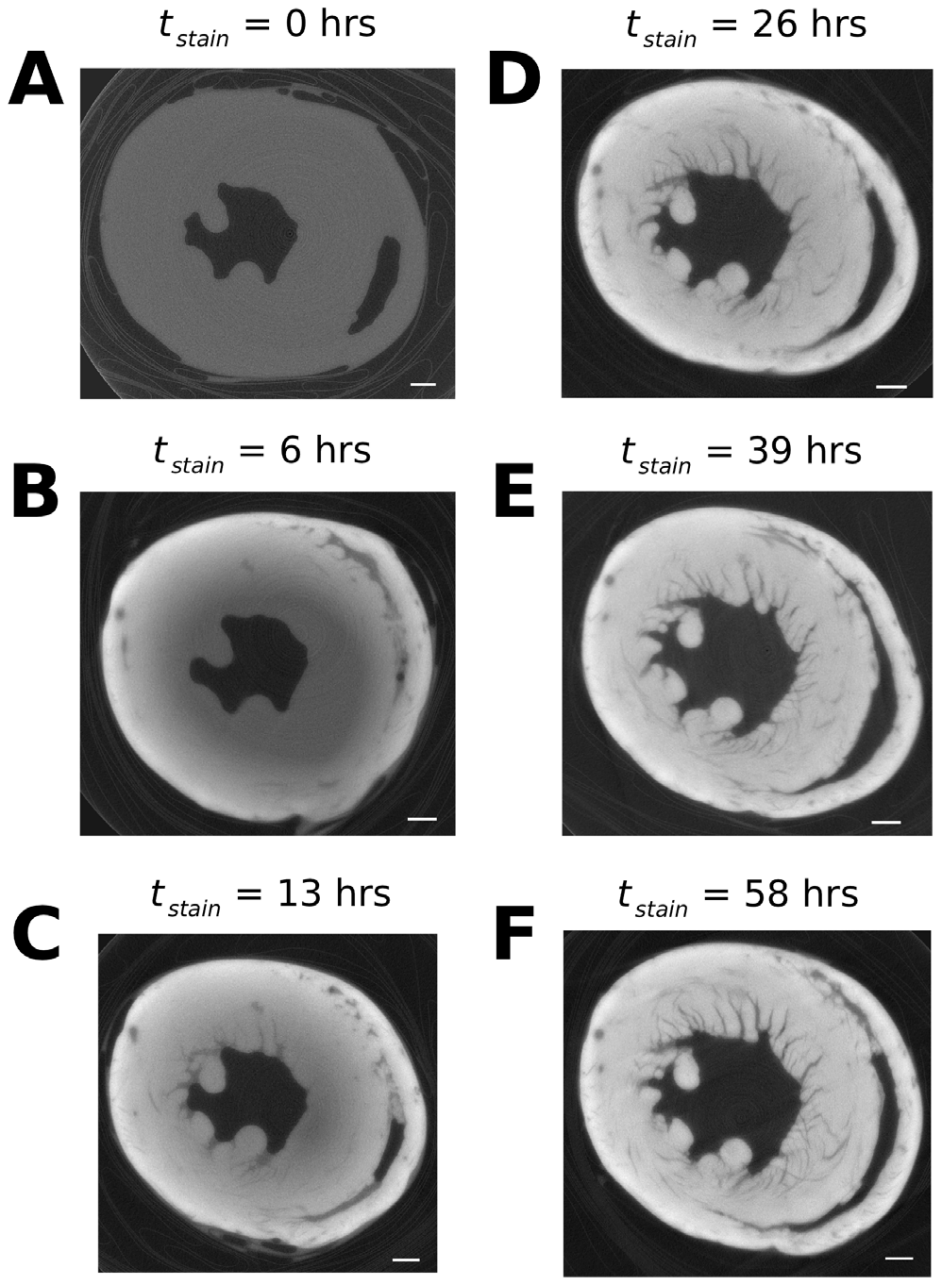
Reconstructed virtual XCT transverse slices of the mouse ventricles at varying staining times. A) 0 hours; B) 6 hours; C) 13 hours; D) 26 hours; E) 39 hours; F) 58 hours. The scale bars in the bottom right of each panel are representative of 1 mm. The polythene wrapping and bijou tube, clearly visible in the periphery of panel A, are shown to be of low contrast compared to the stained tissue (panels B–F).

Whole hearts were excised from four adult mice and fixed in phosphate buffered formal saline (4% formaldehyde made up in phosphate buffered saline) for a period of 48 hours. The use of a fixative such as phosphate buffered formal saline allows for sample storage, and reduces the extent of tissue shrinkage associated with the use of contrast agent [18]. The tissue was stained using the procedure outlined in Stephenson *et al.* [11]. The contrast agent was a 3.75% 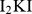 solution made up using 10% neutral buffered formalin. The four samples were placed in the 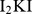 solution at room temperature. After allowing a period of 6–7 hours to stain, the samples were removed in stages from the staining agent and blotted to remove excess solution. Each sample was then wrapped in polythene and inserted into a plastic bijou tube to ensure stability during the scanning process whilst minimising beam attenuation by the sample holder (Fig. 1).

### Scanning Procedure

The specimens were analysed using a Nikon Metris 225/320 kV XCT system in conjunction with a 2K×2K Perkin Elmer detector (1621-16-bit amorphous silicon flat-panel detector with 200* µ*m pixel pitch). This is housed in a customised bay at the Henry Moseley X-ray Imaging Facility at The University of Manchester.

The sample tube containing the plastic-wrapped specimen was fixed to the rotating stage of the XCT system. During the XCT analysis the specimen was rotated over a 360° rotation range for a time period of approximately 24 minutes, collecting 2001 projections per scan with a voxel size 11* µ*m. An accelerating voltage of 50 kV was used, along with a current of 285* µ*A, and an exposure time of 708 ms per projection. In addition the focus was optimized for the low energy spectrum and the system alignment was rechecked in order to optimize resolution.

### Analysis

After image acquisition the data sets were reconstructed using the Nikon Metris XCT-Pro reconstruction software (Metris XT 1.6, version 2.1.3509.24 387, 10 August 2009). No beam hardening correction [19] or noise reduction was applied in order to maintain consistency during our analysis. The reasoning behind this is two-fold: firstly, beam hardening correction is based on the grey level of the image [19]. Since the intensity level varies with staining throughout the study, the correction would be inconsistent between scans. Secondly, the intensity profile through the sample is affected by the uneven staining of the tissue: attempting to obtain a flat intensity profile by applying beam hardening correction therefore defeats the purpose of quantifying the accumulation of contrast agent over time.

Following reconstruction, datasets were partially aligned in 3D such that the apico-basal axis lay parallel with the 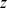-axis (i.e. perpendicular to the transverse plane). 2D transverse slices were then extracted midway through the ventricles for analysis. Due to varying sample placement between scans, it was necessary to ensure that the same region was compared in each dataset. Slice location was noted with respect to the top of the atria and bottom of the ventricles; using this information we were able to identify approximately the same slice in each dataset. This approximation was then verified by identifying distinguishing features (e.g. papillary muscles) to ensure the same region was compared.

**Figure 2:**
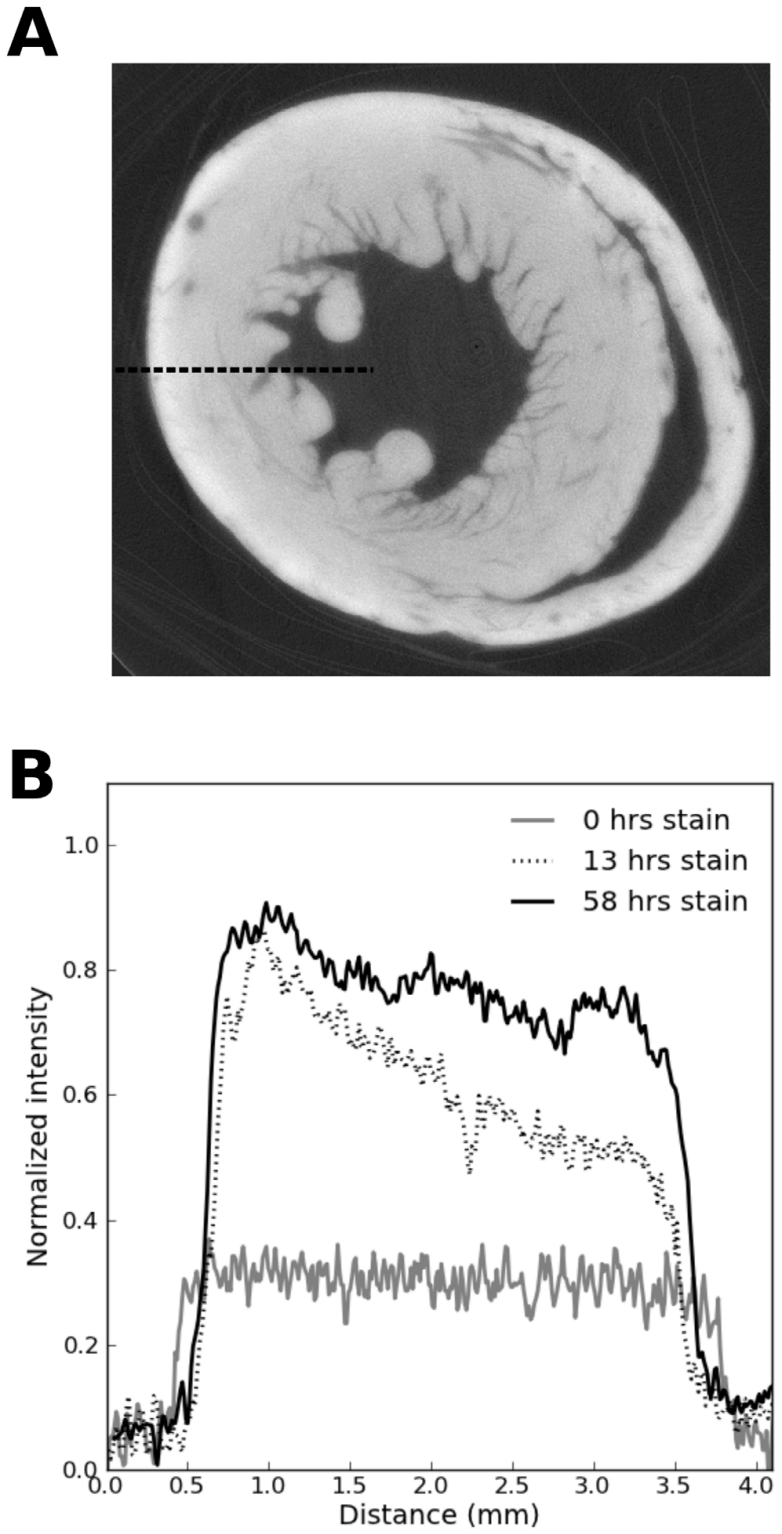
Example tomographic slice with intensity profile across the ventricular wall. A) Transverse tomographic slice through the ventricles with dashed line indicating the sampling location for the intensity line profile. B) Intensity line profiles for 0 hours, 13 hours and 58 hours incubation time.

**Figure 3:**
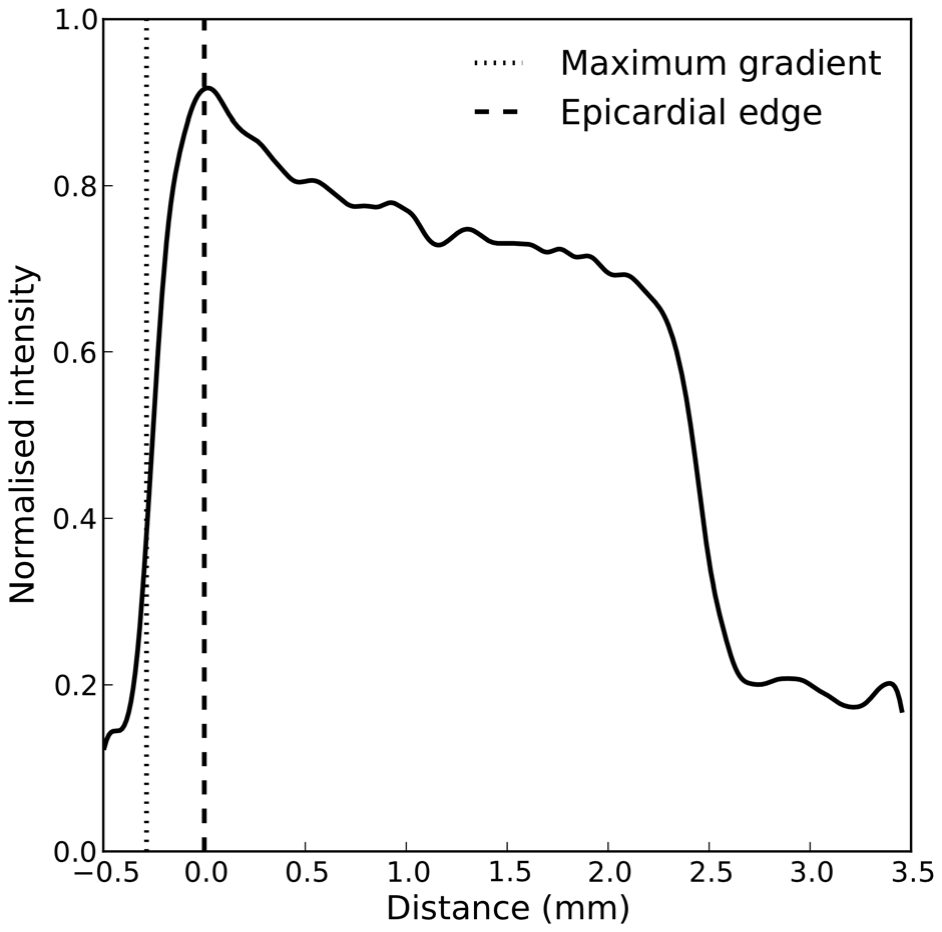
Adopted method for locating the epicardial edge. First the line profile is filtered with a Gaussian kernel (black line; 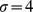) in order to reduce noise. The maximum intensity gradient is subsequently detected (dotted line). The epicardial edge is then defined as the next point (towards the endocardium) at which 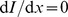 (dashed line).

Once alignment was complete, a line profile of intensity was measured with a width of 5 pixels (taking an average to reduce the effects of noise) from epicardium to endocardium, as Fig. 2 illustrates. The epicardial edge was detected using a three step process, as shown in Fig. 3. 1) The line profile was filtered with a Gaussian kernel (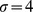) in order to reduce noise, 2) the maximal change in intensity of the line profile 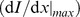 was located, 3) the epicardial edge was defined as the next point towards the endocardium where 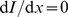. The line profile was then discretized into twenty 0.1 mm segments starting from the epicardial edge. Each segment was assigned an intensity value 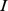 based on the average value of the pixels within the segment, and a value of distance 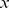 corresponding to the distance between the epicardial edge (0 mm) and each segment and finally a value of 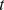 which corresponded to incubation time.

**Figure 4:**
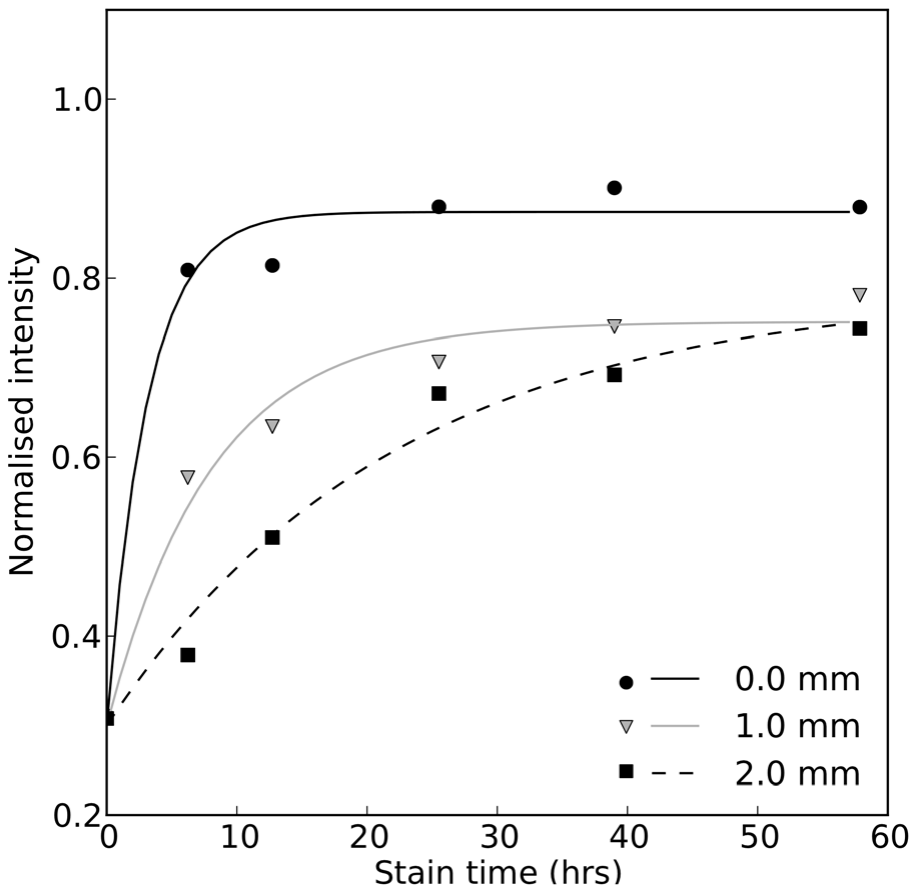
Time course of intensity during the staining process for different points within the tissue. The experimental data is represented by the data points, with fitted curves of the form given in equation 1.

These values were used to analyse the time required to adequately stain tissue at various locations within the ventricular wall. Intensity was plotted against time for various locations (see Fig. 4) within the tissue and fitted with curves of the form:
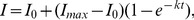
(1)


using MatLab's *fminsearch* function. This formulation assumes that the unstained tissue (i.e. 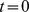) had an initial intensity 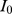 which exponentially tended towards a maximal value of intensity 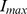 with stain time. We defined optimal staining time as the minimum time required for the tissue to reach its saturated value of intensity. The desirable level of saturation is likely to depend upon the intended application; therefore, we defined three levels of saturation in the ventricles based upon the ratio of 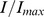 (90%, 95%, 99%) and calculated the optimal staining time accordingly.

## Results

Fig 1. shows the reconstructed slices of the ventricles throughout the staining process. The intensity level of the grey-scale images is shown to increase quicker in the epicardium than the endocardium; since the intensity is related to the amount of 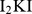 accumulation, this indicates the epicardium accumulates 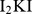 quicker than the endocardium. This is likely due to the fact that the epicardium is in direct contact with the contrast agent and thus stains quicker than other areas. Whilst it is possible that 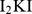 solution could enter the chambers of the heart and stain the endocardium directly, the results indicate that the endocardium took longer to stain, suggesting that contrast agent reached this area primarily by diffusion through the myocardium.

The intensity of each segment was plotted over time and a curve was fitted according to equation 1, as shown in Fig. 4. Representing the data in this way illustrates how the increase in intensity, and thus the accumulation of 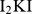, is slower at more distant points within the tissue. The time required to optimally stain each segment of tissue therefore increases with distance into the sample.

**Figure 5:**
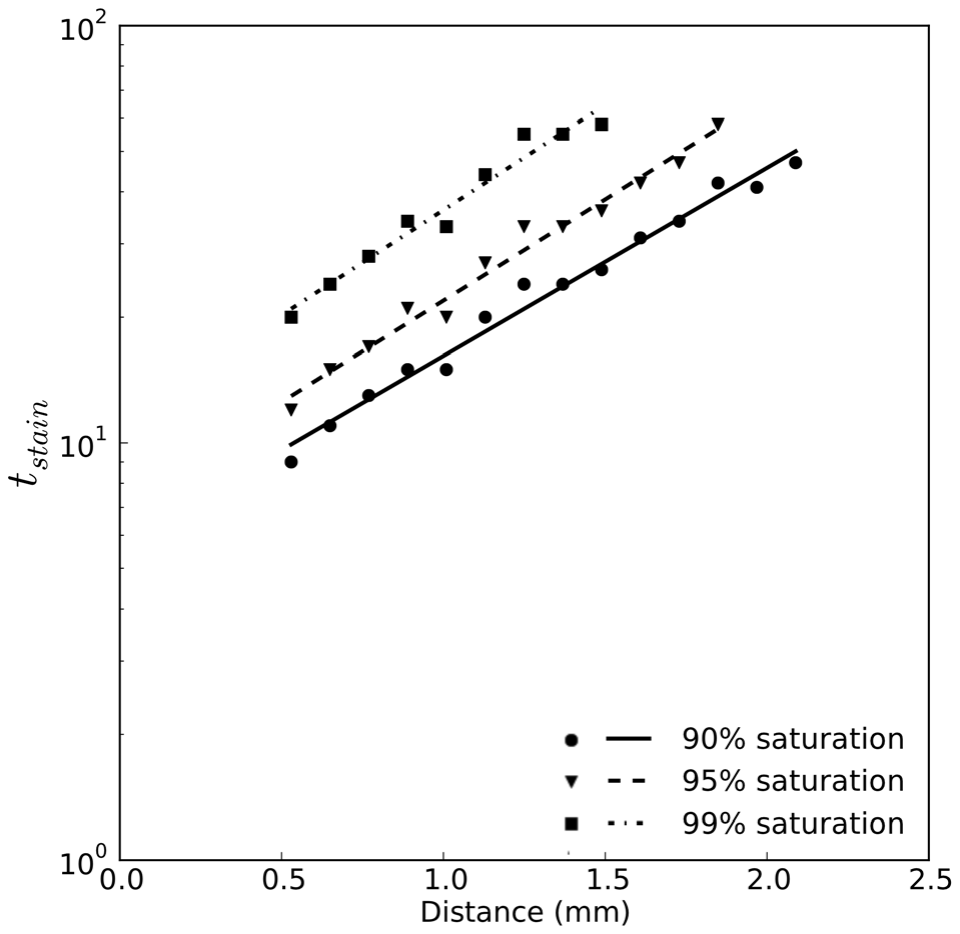
Semi-logarithmic plot of the required staining time for different levels 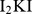 saturation as a function of distance. Curves are fitted according to equation 2 and with fitted coefficients given in Table 1.

Based on the fitted curves, we measured the points at which 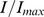 was equal to 90%, 95% and 99%, giving an indication of the optimal staining time throughout the myocardium. Due to the effects of beam hardening, 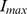 is highest at the epicardium and decreases towards the endocardium. Therefore, taking the ratio of 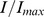 as a marker of tissue stain saturation removed bias due to beam hardening. Having measured these quantities for all values of 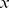 we fitted an equation of the form:
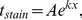
(2)where 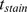 is the required staining time and 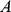 and 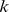 are constants to be fitted for the three saturation values. Fig. 5 shows the result of this fitting process in the form of a semi-logarithmic plot, with fitted values 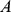 and 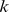 given in Table 1.

**Table 1:** Fitted coefficients for calculating staining times for a given saturation level.

Saturation Level	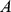	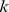
90%	5.72	1.04
95%	7.17	1.12
99%	11.3	1.16

Coefficients 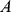 and 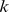 can be used in conjunction with equation 2 to obtain optimal staining time for an arbitrary location within the tissue.

## Discussion

As shown in Fig. 2B, as the staining time increases, the line profile of intensity across the tissue becomes flatter. This indicates a more uniform optical density, which would be expected for homogeneous tissue. If the line profile is not flat, this would indicate an incomplete staining process, as parts of the tissue contain more contrast agent than others. As shown in Fig. 5, the required staining time is exponentially dependent on the distance into the tissue (since the scale is semi-logarithmic a straight line plot is obtained).

To calculate the optimum staining time the values shown in Table 1 can be used in conjunction with equation 2 to determine the time needed to achieve 90%, 95% and 99% saturation at an arbitrary distance into the tissue. For small tissue samples, such as the mouse hearts used in this study, it is possible to achieve a saturation of 99%. This is ideal for segmentation, as a uniform intensity is obtained which facilitates the use of automated algorithms, such as region-growing. For thicker tissue samples a saturation level of 99% may not be practically obtainable; therefore parameters for equation 2 have also been given for 95% and 90% saturation. As stated, it can be seen that in our experiments the diffusion process occurred from the epicardial surface towards the endocardium. Due to the exponential relationship between staining time and tissue thickness it may be necessary to stain larger samples from both the epicardial and endocardial surfaces simultaneously, which would allow twice the amount of tissue to be stained in the same amount of time.

## Limitations

The effect of time upon contrast agent diffusion is one factor affecting contrast of XCT images; other controls such as the concentration of the contrast agent, species and specimen size need to be quantified in further studies to fully understand staining conditions for optimal XCT contrast. Other controllable factors include temperature, contrast agent and solvent used, and are summarised in Table 2.

**Table 2:** Summary of controllable factors and their values used in the study.

Controlled variable	
Species	Mouse
Fixative	Phosphate buffered formal saline
Contrast agent	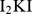
Contrast agent concentration	3.75%
Contrast agent solvent	Formalin (10%)
Temperature	Room temperature
X-ray target	Tungsten
X-ray accelerating voltage	50 kV
Projections	2001

It has been shown that 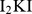 concentration significantly impacts upon scan results [11], [12], [17] and is also likely to affect the incubation time required to successfully stain a soft tissue sample. In our study we chose a constant concentration suitable for imaging smaller hearts [11]. Whilst this has been essential for maintaining a manageable sample size, the effect of concentration on the staining process remains to be fully evaluated.

This study also concentrates only on mouse tissue, with results being extrapolated to cardiac tissue of other animals. Since tissue structure [20]–[23] and density [24]–[26] have only a small amount of variation across species, 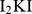 can be expected to stain cardiac tissue in a similar way across species.

## Conclusions

Contrast-enhanced XCT is a technique which can be used to image the whole heart with high resolution compared with other imaging techniques such as MRI. The use of contrast agent allows for detailed structures such as the cardiac conduction system and fibre orientation to be resolved using XCT-based imaging analysis [27]. The quality of results from contrast-enhanced XCT depend greatly upon the staining process, in particular the length of time samples are stained. In this study we have characterised and quantified this process in mouse hearts, and derived an empirical relationship between required incubation time and sample thickness. This relationship can be used as a guide for future contrast enhanced XCT studies of cardiac tissue.
